# Algorithm development for corticosteroid management in systemic juvenile idiopathic arthritis trial using consensus methodology

**DOI:** 10.1186/1546-0096-10-31

**Published:** 2012-08-29

**Authors:** Norman T Ilowite, Christy I Sandborg, Brian M Feldman, Alexi Grom, Laura E Schanberg, Edward H Giannini, Carol A Wallace, Rayfel Schneider, Kathleen Kenney, Beth Gottlieb, Philip J Hashkes, Lisa Imundo, Yukiko Kimura, Bianca Lang, Michael Miller, Diana Milojevic, Kathleen M O’Neil, Marilynn Punaro, Natasha Ruth, Nora G Singer, Richard K Vehe, James Verbsky, Amy Woodward, Lawrence Zemel

**Affiliations:** 1 Children’s Hospital at Montefiore, Albert Einstein College of Medicine, Bronx, NY, USA; 2 Stanford University School of Medicine, Palo Alto, CA, USA; 3 The Hospital for Sick Children, University of Toronto, Toronto, Ontario, CA; 4 Cincinnati Children’s Hospital Medical Center, Cincinnati, OH, USA; 5 Duke University Medical Center, Durham, NC, USA; 6 Seattle Children’s Hospital, Seattle, WA, USA; 7 Steven and Alexandra Cohen Children’s Medical Center of New York, New Hyde Park, NY, USA; 8 Cleveland Clinic Lerner Medical School, Cleveland, OH, Shaare Zedek Medical Center, Jerusalem, Israel; 9 Children’s Hospital of New York, New York, NY, USA; 10 Hackensack University Medical Center, Hackensack, NJ, USA; 11 IWK Health Center, Dalhousie University, Halifax, NS, USA; 12 Children’s Memorial Hospital, Chicago, IL, USA; 13 University of California San Francisco Medical Center, San Francisco, CA, USA; 14 University of Oklahoma University Health Sciences Center, Oklahoma City, OK, USA; 15 Texas Scottish Rite Hospital for Children, Dallas, TX, USA; 16 Medical University of South Carolina, Charleston, SC, USA; 17 University Hospitals/Case Medical Center, Cleveland, OH, USA; 18 University of Minnesota, Minneapolis, MN, USA; 19 Medical College of Wisconsin, Milwaukee, WI, USA; 20 Vanderbilt University Medical Center, Indianapolis, IN, USA; 21 Connecticut Children’s Medical Center, Hartford, CT, USA

## Abstract

**Background:**

The management of background corticosteroid therapy in rheumatology clinical trials poses a major challenge. We describe the consensus methodology used to design an algorithm to standardize changes in corticosteroid dosing during the Randomized Placebo Phase Study of Rilonacept in Systemic Juvenile Idiopathic Arthritis Trial (RAPPORT).

**Methods:**

The 20 RAPPORT site principal investigators (PIs) and 4 topic specialists constituted an expert panel that participated in the consensus process. The panel used a modified Delphi Method consisting of an on-line questionnaire, followed by a one day face-to-face consensus conference. Consensus was defined as ≥ 75% agreement. For items deemed essential but when consensus on critical values was not achieved, simple majority vote drove the final decision.

**Results:**

The panel identified criteria for initiating or increasing corticosteroids. These included the presence or development of anemia, myocarditis, pericarditis, pleuritis, peritonitis, and either complete or incomplete macrophage activation syndrome (MAS). The panel also identified criteria for tapering corticosteroids which included absence of fever for ≥ 3 days in the previous week, absence of poor physical functioning, and seven laboratory criteria. A tapering schedule was also defined.

**Conclusion:**

The expert panel established consensus regarding corticosteroid management and an algorithm for steroid dosing that was well accepted and used by RAPPORT investigators. Developed specifically for the RAPPORT trial, further study of the algorithm is needed before recommendation for more general clinical use.

## Background

One of the major challenges in designing and analyzing clinical trials in rheumatology is the management of background therapy, especially corticosteroids. This report describes the process whereby investigators in one study; the ***Ra***ndomized ***P***lacebo ***P***hase Study ***o***f ***R***ilonacep***t*** in Systemic Juvenile Idiopathic Arthritis ***T***rial (RAPPORT), used consensus methodology to design an algorithm to standardize changes in corticosteroid dosing during the study. RAPPORT is a study of the efficacy and short-term safety of the IL-1 inhibitor rilonacept in systemic JIA (SJIA), which utilized 20 sites from the Childhood Arthritis and Rheumatology Research Alliance (CARRA), and was funded by the National Institute of Arthritis, Musculoskeletal and Skin Diseases (NO1 AR 700015).

Corticosteroid therapy is traditional treatment for patients with SJIA who have systemic manifestations [1]. It is usually initiated soon after diagnosis because rapid onset is often necessary for stabilization of severe disease. Protocol limitations regarding background corticosteroid therapy directly impact study feasibility and results in multiple ways. First, a major problem in the design of clinical trials with corticosteroid background therapy is the diversity of practice standards among treating rheumatologists in the use of corticosteroids potentially leading to lack of acceptance of the protocol. Second, the estimation of a change in disease activity can only be interpreted in the light of the use, absence or change in dose of corticosteroids. Third, maximal corticosteroid tapering is desirable during the course of the study to minimize exposure to high doses of corticosteroids and associated toxicity. Lastly, life-threatening complications of SJIA, e.g. serositis and macrophage activation syndrome (MAS, also termed hemophagocytic lymphohistiocytosis [HLH]) can be unmasked by corticosteroid tapering [2]; therefore, corticosteroid regimens must minimize the risks of these complications occurring or worsening during SJIA trials. Presently, however, there are no clinical studies or trials upon which to develop evidence-based guidelines regarding corticosteroid therapy in SJIA [3].

RAPPORT utilized a randomized placebo phase study design which determined the efficacy of study drug based on the premise that subjects starting active drug sooner would, on average, respond sooner than subjects starting active drug later [4]. This study design minimized time on placebo in a systemically ill population (4 weeks in subjects randomized to placebo) while capturing responses to study drug 2–12 weeks after randomization. The primary efficacy end point was time to response using a tri-partite composite definition of response specifically designed for the clinical profile of prospective subjects in this trial, i.e., systemically ill children, often on corticosteroids, who have at least 2 joints with active arthritis. Response was defined as: 1) achieving the ACR Pediatric 30 definition of improvement (30% improvement in at least 3 of 6 core criteria without worsening of ≥30% in more than 1 criterion [5]), 2) remaining afebrile for 2 weeks, and 3) successful taper of corticosteroids by 10% for subjects taking corticosteroids, while sustaining all response criteria for at least 2 weeks (until the next visit). Initiating or increasing the dose of corticosteroids during the follow-up period categorized a subject as a non-responder. Clearly, changing corticosteroid dosing, either by starting, increasing or tapering was integrally related to the primary endpoint. Therefore, the use of corticosteroids in this trial had to be explicitly defined and standardized.

We describe the consensus methodology used to develop criteria for starting, increasing or tapering corticosteroids in the context of the RAPPORT trial. Our goals were to minimize the confounding influence of corticosteroids on interpretation of efficacy and safety data, minimize exposure of subjects to corticosteroids, maximize participant safety, as well as increase acceptability to the participating investigators/treating physicians, parents and patients.

## Methods

In order to enhance trial design acceptance so as to improve patient recruitment and retention, RAPPORT site principal investigators (PIs) joined authorities in SJIA making up the expert panel to develop standardized treatment algorithms for RAPPORT. Indications for starting, increasing and tapering corticosteroids were evaluated. The consensus process used the modified Delphi Method, which includes a combination of written questionnaires (via the on-line survey tool, SurveyMonkey™) and a one day face-to-face consensus conference. The modified Delphi process has been widely used in pediatric and adult rheumatology to develop guidelines for diagnostic criteria, disease activity and severity scores and treatment [6-10]. This method facilitates development of consensus in situations with a limited evidence base, providing consensus-based group processes that lead to high-level decision making. In contrast to the usual Delphi questionnaire process which includes distribution to a large group of experts, the on-line questionnaire was completed by a select group of experts to reach consensus for this specific study (RAPPORT) using the actual individual investigators performing the study. For this study the definition of the consensus level was set at ≥ 75% agreement. For items deemed essential but when consensus on critical values was not achieved, simple majority vote drove the final decision.

### Preconference questionnaire

The initial questionnaire focused on indications for initiating, increasing and tapering corticosteroid therapy. Each expert’s specific approach to use of corticosteroids in SJIA was queried, including corticosteroid dosing and delivery routes, frequency, duration of treatment, and general approach to tapering. The on-line survey was comprised of 2 sections: 1) reasons for initiating or increasing corticosteroids (43 specific elements) and 2) criteria for tapering corticosteroids (12 elements). The principal investigators for RAPPORT (CS, NI) identified areas of agreement and areas of difference among the respondents. The results of the e-mail questionnaire were distributed to the members of the expert panel prior to the face to face meeting.

### Full day consensus conference

The expert panelists first reviewed the current literature regarding the use of corticosteroids in SJIA. The PIs presented abstracted data derived from a systematic review of the literature. The PIs also presented the results of the on-line questionnaire in order to identify potential omissions of clinically important variables. Based on data from the on-line questionnaire and identified omissions, items of consensus were either rejected or endorsed and any disagreements discussed in a structured, nominal group technique (NGT) format [11]^.^ Panelists were then sorted into 2 nominal groups by the PIs (NI, CS) and consensus methodology experts (EG, CW) based on optimal group interactions for consensus (no more than 12 participants, distributed by age, perceived expertise, personal characteristics) [11,12]. Each group separately discussed the same items and areas of agreement were endorsed. Disagreements were discussed, again using modified NGT, which allows structured interactive discussion while minimizing potential negative aspects of small group interactions such as group-think, effects of status or style, and other observed behaviors [13,14]. Initially each item was reviewed and opinions elicited from each panelist. Structured discussion was based on a round-robin (taking turns in raising or further discussing issues) or similar process, followed by voting. If consensus was achieved (arbitrarily defined as ≥ 75% agreement), the next item was considered in similar fashion. When consensus was not achieved from the initial discussion, the same item was again discussed by all nominal group members in a round-robin process until consensus was either achieved or deemed deadlocked. Following the nominal group meetings, the entire panel convened to review the merged results from the two groups in order to increase the robustness of the process and to identify areas of bias or polarization within the smaller groups. Areas of consensus were identified and areas of difference discussed. Deadlocked items from each group were then discussed. For those items that were deemed essential but where ≥ 75% consensus was not achieved, majority vote was used to define acceptable levels of consensus. In rare cases, the expert panel decided to delegate the decision to the RAPPORT study PIs (CS, NI). Using all the consensus-developed data and criteria, two algorithms (one for starting or increasing corticosteroids, the other for tapering) were developed and circulated among the panel by e-mail for approval.

## Results

Twenty RAPPORT investigators and 4 topic experts (2 in consensus methodology [EG, CW], one in randomized placebo phase study design [BF], and one in MAS [AG]) participated in the on-line questionnaire and/or the one day consensus conference and constituted the expert panel. One consensus methodology expert (CW) was also a site PI. The non-physician consensus methodology expert (EG) did not participate in consensus voting. Twenty of 23 individuals (91%) responded to the on-line questionnaire and 19 of 23 (83%) participated in the face-to-face consensus conference. A list of questions and level of consensus achieved for the on-line questionnaire are provided in Table 1.

**Table 1:** Questions and results from on-line questionnaire

**QUESTIONS**	**CONSENSUS (%)**
Question 1. What do you use as criteria for initiating treatment with corticosteroids with the following cardiopulmonary disease involvement? Assume that NSAIDs have not been effective.	
a. Presence of symptomatic pericarditis?	Yes (100%)
b. Presence of symptomatic myocarditis?	Yes (100%)
c. Presence of asymptomatic myocarditis (imaging only)?	Yes (84%)
d. Presence of symptomatic pleural effusion?	Yes (100%)
e. Presence of symptomatic pneumonitis?	Yes (100%)
Question 2. Would rash alone be an indication for corticosteroids?	No (95%)
Question 3. Would you consider fever alone an indication for corticosteroids?	
a. For 6 of last 10 days?	Yes (75%)
b. For 8 of last 14 days?	Yes (92%)
Question 4. Would severe fatigue (defined as inability to attend school or participate in regular activities) alone be an indication for corticosteroids? Assume no other causes identified.	No (74%)
Question 5. Would you consider anemia alone to be an indication for corticosteroids?	
a. For Hgb less than 7 g/dl?	Yes (100%)
b. For Hgb 7–9 g/dl?	Yes (54%)
Question 6. Would anorexia alone be an indication for corticosteroids? Assume no other causes identified.	No (95%)
Question 7. Would weight loss alone be an indication for corticosteroids? Assume no other causes identified.	No (74%)
Question 8. Would low albumin alone be an indication for corticosteroids? Assume no other causes identified.	No (95%)
Question 9. Would any of the findings of MAS below alone be an indication for corticosteroids?	
a. CNS dysfunction?	Yes (85%)
b. Purpura, easy bruising, mucosal bleeding?	Yes (85%)
c. Increasing ferritin?	No (75%)
d. Decreasing ESR?	No (78%)
e. Increasing d-dimers?	No (71%)
f. Decreasing fibrinogen?	No (61%)
g. Decreasing WBC?	No (55%)
h. Decreasing platelets?	Yes (58%)
i. Increasing LFTs?	Yes (50%)
j. Hepatomegaly?	No (84%)_
Question 10. For those questions above regarding incomplete MAS to which you answered “no” (i.e. the finding alone would not be an indication for corticosteroids), would any combination of the above findings be an indication for starting corticosteroids?	
a. Increasing ferritin ?	Yes (59%)
b. Decreasing ESR?	Yes (59%)
c. Increasing d-dimers?	Yes (59%)
d. Decreasing fibrinogen?	Yes (47%)
e. Decreasing WBC?	Yes (41%)
f. Decreasing platelets?	Yes (35%)
g. Increasing LFT’s?	Yes (53%)
h. Hepatomegaly?	Yes (18%)
Question 11. This question refers to questions 1–9 above. For those questions to which you answered “no” (i.e. the finding alone would not be an indication for corticosteroids), would any combination of the above findings be an indication for starting corticosteroids?	
a. Weight loss	Yes (69%)
b. Anemia	Yes (63%)
c. Fever	Yes (63%)
d. Rash	Yes (60%)
e. Hypoalbuminemia	Yes (50%)
f. Fatigue	Yes (44%)
g. Asymptomatic pericarditis	Yes (31%)
h. Anorexia	Yes (31%)
i. Asymptomatic pleural effusions	Yes (19%)
Question 12. Would you require complete resolution by imaging of pericarditis, myocarditis, pleural effusion and/or pneumonitis before tapering corticosteroids?	No (85%)
Question 13. Is there a lower limit of corticosteroid dose beyond which you would not continue to taper (i.e. a maintenance dose)?	No (79%)

### Consensus criteria for initiating or increasing corticosteroids

The first set of questions on the questionnaire referred to criteria for initiating treatment with corticosteroids or increasing the current dose of corticosteroids. Table 2 shows both those reaching consensus in the on-line questionnaire, and those reaching consensus during the face-to-face conference that did not meet consensus in the questionnaire. All respondents agreed symptomatic pericarditis, pleural effusions, pneumonitis or asymptomatic or symptomatic myocarditis, and MAS were indications to start or increase corticosteroids. Other stand-alone criteria or symptoms that did not reach consensus as an indication for starting or increasing corticosteroids were fever, rash, severe fatigue, anorexia, weight loss, increasing synovitis and low albumin.

**Table 2:** Clinical features indicating active SJIA requiring initiation or increase of corticosteroids

	**On Line**	**Questionnaire**		**Face-to-Face Conference**
	**Consensus reached (%)**	**Increase cortico-steroids?**	**Consensus not reached**	Consensus*
Increase corticosteroids in the presence of				
Symptomatic pericarditis	Yes	Yes		Endorsed
Symptomatic myocarditis	Yes	Yes		Endorsed
Asymptomatic myocarditis (imaging only)	Yes	Yes		Endorsed
Symptomatic pleural effusion	Yes	Yes		Endorsed
Symptomatic pneumonitis	Yes	Yes		Endorsed
Fever for 6 of last 10 days	Yes	Yes		Changed to “No”
Fever for 8 of last 14 days	Yes	Yes		Changed to “No”
Anemia, Hgb <7 g/dl	Yes	Yes		Refined as “No” if ≥ 6.5 and asymptomatic
Anemia, Hgb 7–9 g/dl			X	No if ≥ 6.5 g/dl and symptomatic
SJIA rash alone	Yes	No		Endorsed
Anorexia alone	Yes	No		Endorsed
Low albumin alone	Yes	No		Endorsed
Severe fatigue alone			X	No
Weight loss alone			X	No
Worsening synovitis*	Not evaluated			No

*Items from on-line questionnaire reaching consensus were reviewed and either endorsed or changed based on discussion during face-to-face consensus process.

#### Anemia

Seventy-six percent of respondents agreed that severe anemia alone was an indication for starting or increasing corticosteroids. Of those who responded that anemia alone was an indication for starting or increasing corticosteroids, all considered hemoglobin less than 7 g/dl and over 53% thought that 7–9 g/dl warranted therapy. More granular discussion in the face-to-face conference led to consensus that corticosteroid dosing should be initiated or increased for symptomatic hemoglobin below 6.5 g/dl.

#### MAS

All investigators agreed that the presence of MAS was an absolute indication for increasing or initiating corticosteroids. However, the criteria for diagnosing MAS differed among investigators reflecting the lack of validated criteria. Particular attention was focused on preliminary criteria for MAS developed by Ravelli et al. [15] and the Histiocyte Society’s revised HLH criteria [16]. The Ravelli criteria for MAS were examined closely during the face-to-face conference with the total group together. Details regarding the database used to develop the criteria (including the lack of available ferritin data), its preliminary nature and lack of validation were presented to the entire panel. The entire expert panel expressed concern that improving SJIA might be erroneously misclassified as MAS using the Ravelli criteria in RAPPORT. The panel achieved consensus that the 2004 HLH criteria [16] as reproduced in Table 3, were appropriately stringent to identify subjects who needed to start or increase their corticosteroid dose.

**Table 3:** **Hemophagocytic lymphohistiocytosis (HLH) criteria **[16]


**Criteria (5 or more of the following)**	
Fever (last 4/7 days)	Hemophagocytosis in tissue
Splenomegaly	TG ≥265 mg/dl
Bi-cytopenia (affecting ≥ 2 of 3 cell lineages) Platelets < 100,000/ml Neutrophils <1000/ml, Hgb < 9 g/dl	Ferritin ≥500 mcg/L
Low NK (natural killer cells) activity	Elevated CD25 (sIL-2R) ≥ 2,400 U/ml
Fibrinogen ≤ 1.5 g/L	

#### Incomplete MAS

The panel similarly agreed that signs and symptoms that indicated early or impending MAS (termed incomplete MAS) would be treated by initiating or increasing corticosteroids to abort the development and resulting morbidity of complete MAS. As validated diagnostic criteria for incomplete MAS do not currently exist, the on-line questionnaire queried respondents about what parameters indicating “incomplete” or “impending” MAS would be important stand-alone diagnostic criteria and what combinations of clinical and laboratory features would warrant initiating or increasing corticosteroids. CNS dysfunction and purpura/bruising/bleeding easily reached consensus, but other responses varied widely (see Table 1, Questions 9 and 10). Therefore, based upon the complexity and clinical safety importance of this issue, the expert panel at the face-to-face consensus conference decided to discuss this important issue of managing incomplete MAS as an entire group. The first decision task was ranking clinical and laboratory features in order of importance for defining incomplete MAS (Table 4). D-dimer levels were removed by majority vote from the criteria based on the clinical experience of the group that d-dimer levels are often elevated in active SJIA without other evidence of incomplete MAS. Conference participants then reached consensus regarding the laboratory values important for diagnosis of incomplete MAS and agreed that one Major and one Minor or three Minor criteria were required for diagnosis (Table 4).

**Table 4:** Ranking of factors for defining incomplete MAS*

**Finding**	**Ranking (mean)**	**Level**	**Definition**
CNS dysfunction	N/A	Major	Present
Bleeding/easy bruising/purpura	N/A	Major	Present
Ferritin	5.86	Minor	>5000 ng/ml & increasing
Platelets	5.24	Minor	<150,000/ml or < LLN for lab
PT (INR)	3.52	Minor	>1.2
Fibrinogen	3.52	Minor	<LLN
WBC	3.33	Minor	<LLN
LFT’s	3.10	Minor	>2 XN
d-dimer	3.10	Rejected	

*1 major and 1 minor or 3 minor are required to meet criteria for diagnosis of incomplete MAS.

LLN lower limit of normal; XN times normal.

### Corticosteroid dosing

There was variable consensus in the on-line questionnaire regarding dosing regimens for increasing or initiating corticosteroids, except for treatment of MAS with an initial regimen of high-dose IV methylprednisolone pulse (30 mg/kg/d for 1–3 days) followed by daily corticosteroids. In the presence of symptomatic pericarditis and myocarditis, 80% respondents agreed on IV methylprednisolone pulse therapy, then daily oral dosing. The oral doses chosen by the respondents varied widely, was dependent upon the severity of the manifestation, and consensus was not reached on specific corticosteroid dosing.

### Consensus criteria for tapering corticosteroids

At each RAPPORT study visit, the protocol requires the investigator to determine if the subject meets criteria for tapering corticosteroids (if applicable). The criteria identified by each nominal group were different. Therefore, all criteria were re-evaluated by the entire group even in areas of consensus to develop a single algorithm for corticosteroid taper. Consensus was facilitated by in-depth discussion of the performance and implications of the randomized placebo phase study design (led by BF), and how overly aggressive corticosteroid taper might be unsafe and result in disease flares, introducing an important confounder and impact study results. On the other hand, if the corticosteroid taper was too slow, it would result in higher corticosteroid doses than necessary and mask differences in response between the two study arms. The laboratory and clinical criteria required for corticosteroid taper in this trial as determined by consensus of the entire panel at the face-to-face meeting are shown in Table 5.

**Table 5:** Clinical and laboratory criteria for corticosteroid taper

**Criteria**	**Required for steroid taper:**	**No increase or taper of corticosteroids**
Ferritin	≤2500 mcg/l	N/A
Platelets	≤800,000/ml	>800,000/ml
Fibrinogen	>LLN	N/A
INR	≤1.2	N/A
WBC	>LLN	N/A
LFTs	≤2x ULN	N/A
Hemoglobin	≥ 7.5 g/dl	≤7.5 g/dl
Fever	≤2 days in last 7	≥3 days in last 7
Physical functioning*	Acceptable	Poor

*As determined by investigator (for example, non-ambulatory, not attending school, anorectic, losing weight).

LLN lower limit of normal; N/A not applicable.

Additionally, the panel decided that three options (taper, increase, or maintain steroid dose) would optimize retention of subjects in the study rather than forcing a taper under potentially unsafe clinical conditions that might lead to disease flare requiring an increase in corticosteroids. The inclusion of a global assessment of physical functioning by the investigator was identified as an important safety check, reflecting the clinical judgment of the physician caring for the patient. However, the following isolated clinical and laboratory features were deemed not useful in determining taper, including weight change, rash, fatigue, anorexia, fever, white blood count, hypoalbuminemia, hemoglobin level, platelet count, ESR or CRP. In addition, 85% of respondents agreed complete resolution of pericarditis, myocarditis, pleural effusion and/or pneumonitis documented by imaging was not required before tapering corticosteroids. The rate of taper determined by consensus was 10% of the current daily dose every two weeks. The expert panel delegated the decision on how to manage the corticosteroid taper to the PIs (NI, CS).

**Figure 1:**
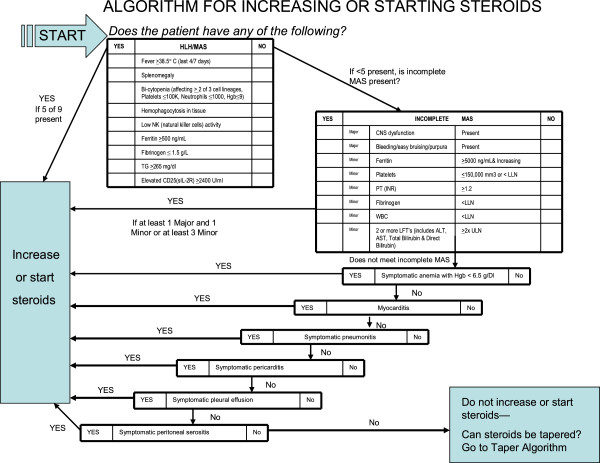
Algorithm for increasing or starting steroids.

**Figure 2:**
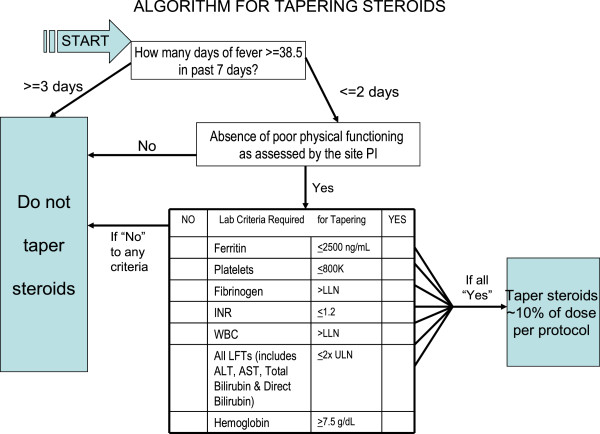
Algorithm for tapering steroids.

In summary, the consensus conference developed three options regarding corticosteroid dosing during the efficacy phase of RAPPORT (see Figure 1 and 2).

### To start or increase corticosteroids

Subjects must either have complete MAS using the Histiocyte Society’s HLH criteria [16], or incomplete MAS defined as the presence of one major (CNS dysfunction or bleeding/bruising/purpura) and one minor criteria, or 3 minor criteria. Additional stand-alone criteria for starting or increasing corticosteroids included symptomatic anemia with hemoglobin <6.5 g/dl, symptomatic serositis unresponsive to NSAIDs, or symptomatic pneumonitis.

### Tapering of corticosteroids

Only occurred if subjects met specified clinical and laboratory criteria and did not have poor physical function as identified by the site principal investigator, hemoglobin < 7.5 g/dl, platelets > 800,000/mm [3] or fever more than 3 of 7 days preceding the study visit. Taper of 10% of the current dose was required every two weeks if tapering criteria were met.

### Corticosteroid dose was left unchanged

For subjects not satisfying the starting, increasing, or tapering criteria.

## Discussion

In the design of any clinical trial, issues of scientific integrity and rigor are balanced against issues of safety, feasibility and logistical applicability. The RAPPORT investigators recognized that some degree of scientific rigor was sacrificed in exchange for enhanced feasibility (improved enrollment and retention of subjects) and safety by allowing variability in corticosteroid dose during the efficacy phase of the study. This approach permitted subjects on higher doses of corticosteroids to be included in the study, in contrast to other JIA trials, as their corticosteroid doses could be decreased by protocol if they responded to study medication, rather than requiring stable higher dose corticosteroids for longer than clinically indicated. This approach also better approximated “real life” challenges of caring for children with SJIA. Additionally, a standardized rate of taper was developed for study drug responders, in order to highlight differences in efficacy between the two treatment arms (those who started active rilonacept initially and those who started active drug 4 weeks later). Because increasing or starting corticosteroids would result in the subject being classified as a non-responder, the panel recognized that the frequency of starting or increasing corticosteroids should be standardized to avoid inadvertently and adversely affecting the retention of subjects in the efficacy phase of the study, while ensuring subject safety.

One key element that improved the ability to reach consensus was the presence of experts present who responded to panel questions and concerns quickly and authoritatively. Specifically, the availability of an expert in the trial design (BF) providing comprehensive information about the impact of starting or increasing corticosteroids on study performance, as well as an expert on the diagnosis of MAS (AG) who shared new data concerning the use of ferritin to distinguish MAS from SJIA flare [17,18], greatly enhanced the consensus discussions and ensured scientific rigor. These data, which are now published [18], showed that in 5/16 SJIA patients with elevated MAS biomarkers (sIL2ra and sCD163), the median ferritin level was 2,950 ng/ml the median level in 11 SJIA patients with normal MAS biomarkers was 179 ng/ml. This suggests that ferritin levels can distinguish MAS from SJIA flare to some degree. Experienced group leaders (EG, CW) ensured that the consensus process was robust and not affected by negative behaviors that can detract from group decision making, such as group-think, effects of status or style, obedience, compliance, conformity, group polarization or minority influence [13,14]. Having site PIs participate in the consensus process as the expert panel enhanced study engagement and “buy-in” regarding the final protocol.

While the modified Delphi NGT process performed well for the majority of the decisions, two major areas required discussion by the entire panel since there was the lack of agreement between the two formal nominal groups. These areas were 1) the determination of criteria for incomplete MAS, as there was little available information on which to base consensus; and 2) determination of criteria for corticosteroid taper. Revisiting the principles of the trial design as a combined group permitted consensus to be achieved. Another unplanned but valuable outcome of this process was development of the first criteria for diagnosis of incomplete MAS, which will require further study and validation.

The expert panel successfully achieved consensus on most items considered despite the highly variable and individualized approach to corticosteroid treatment for SJIA. The consensus resulted in the design of algorithms to standardize changes in corticosteroid dosing that was successfully implemented in RAPPORT and positively accepted by the investigators. The algorithms were designed to be optimal for the RAPPORT clinical trial only and not intended as a guideline for clinical care of children with SJIA outside of the trial setting. In fact, Childhood Arthritis and Rheumatology Research Alliance (CARRA) investigators have recently developed consensus treatment plans for SJIA to be used in clinical practice [19]. While these plans include recommendations on dosing of corticosteroids [15], they do not suggest specific criteria for corticosteroid dose changes. It may be valuable to combine the CARRA guidelines and the RAPPORT algorithms for future rigorous study.

Although RAPPORT enrollment is complete, the results of the study are not yet available to fully evaluate whether development and implementation of corticosteroid algorithms ultimately had a beneficial impact on enrollment or retention of subjects, or the projected impact on study results. However, near universal positive feedback, as assessed on monthly conference calls with site PIs and research coordinators, and an interim analysis of data from 55 subjects identified only 6 errors in applying the algorithm out of 622 monitored visits. This suggest that the use of consensus methodology to drive research protocol development in controversial areas may be a worthwhile model for other studies, particularly pragmatic trials.

## Conclusion

The use of consensus methodology among site investigators and topic specialists can be an effective method for developing plans for the management of concomitant medications in clinical trials.

## Competing interests

N Ilowite (lead author) declares the following competing interests: Honoraria for consulting from Genentech, Novartis, Abbott, Janssen; Regeneron provided study drug and placebo. Y Kimura declares the following competing interests: Honoraria for consulting from Genentech, Novartis. P Hashes declares the following competing interests: Regeneron provided study drug and placebo for a clinical trial in familial Mediterranean fever. All other authors declare that they have no competing interests.

## Authors’ contributions

Drs. NTI and CIS had primary responsibility for the design of the study and writing of the manuscript. All authors contributed to the conception and design of the study, acquisition of data, data analysis and interpretation of data, drafting of manuscript and critical revisions for intellectual content. All authors except Dr. EHG voted in the consensus conference. All authors have read and approved the final manuscript.

## Authors’ information

Supported by a grant from the National Institute of Arthritis, Musculoskeletal and Skin Diseases (NO1 AR 700015) (ClinicalTrials.gov identifier NCT00534495) and by the Arthritis Foundation. B Feldman declares that he is on the Data Safety and Monitoring Boards for drugs from Novartis, Pfizer, and Bristol Myers Squibb, and has research support from Baxter Labs and Bayer.
